# Boosting the Performance of Visible/Near-Infrared Organic Photodetectors via Hole Interface Engineering

**DOI:** 10.3390/nano16110644

**Published:** 2026-05-22

**Authors:** Yijing Fan, Junquan Luo, Lan Liu, Qiao He, Jiahui Lu, Zhimin Shao, Zhensheng Xu, Zhe Liu, Yun Xia, Xuanye Li, Lintao Hou

**Affiliations:** 1 Guangzhou Key Laboratory of Vacuum Coating Technologies and New Energy Materials, Guangdong Provincial Engineering Technology Research Center of Vacuum Coating Technologies and New Energy Materials, College of Physics and Optical Engineering, Siyuan Laboratory, Department of Physics, Jinan University, Guangzhou 510632, China; fyj05@stu2023.jnu.edu.cn (Y.F.);; 2 Analytical and Testing Center, Jinan University, Guangzhou 510632, China; 3 LEDTEEN Opto Semiconductors (Guangzhou) Co., Ltd., Guangzhou 510663, China; 4 State Key Laboratory of Extreme Photonics and Instrumentation, International Research Center for Advanced Photonics, College of Optical Science and Engineering, Zhejiang University, Hangzhou 310027, China

**Keywords:** organic photodetector, interface engineering, hole transport layer, self-assembled monolayer

## Abstract

When poly(3,4-ethylenedioxythiophene) polystyrene sulfonate (PEDOT:PSS) is employed as the hole transport layer in visible/near-infrared photodetectors, the extraction and transport of holes are hindered by the accumulation of the PSS insulating phase at the interface. This accumulation results in an increase in contact resistance and creates a potential barrier for hole injection. This study introduces a self-assembled monolayer, (2-(9H-carbazol-9-yl)ethyl)phosphonic acid (2PACz), to modify PEDOT:PSS, effectively optimizing the interface of the hole transport layer. Such improvements lead to a reduction in recombination losses during charge transfer, a lower dark current, and improved energy level alignment in the device, thereby boosting the performance of visible/near-infrared photodetectors. The fabricated double hole layer photodetector exhibits a low dark current of (1.4 ± 0.6) × 10^−5^ A at −1 V bias and a switching ratio of up to 7.62 × 10^5^ at 0 V bias. The device achieves a responsivity of 0.31 A/W and a high specific detection rate of 3.23 × 10^12^ Jones at a wavelength of 780 nm, which corresponds to the peak responsivity, showcasing enhanced detection capabilities. In comparison to a reference device based on PEDOT:PSS, the response speed, cutoff frequency, and linear dynamic range of the double hole layer device have been enhanced by 400%, 213%, and 81%, respectively, thereby better aligning with practical application requirements. This research presents a novel approach for the development of high-performance organic visible/near-infrared photodetectors.

## 1. Introduction

Visible/near-infrared light, a crucial component of the electromagnetic spectrum, has extensive applications across various fields, including military navigation, optical communications, pollution detection, and meteorology [1,2]. To transform this information into analyzable and usable data, we must rely on a critical intermediary: the photodetector [3,4,5]. Compared to traditional inorganic photodetectors, organic photodetectors (OPDs) offer advantages such as tunable absorption characteristics and a trend towards the development of flexible devices [6,7]. Typical OPDs exhibit a multilayer heterogeneous thin film structure, where the interface layer plays a vital role in regulating energy level matching, suppressing dark current, and optimizing physical contact [8]. However, the performance of OPDs continues to lag behind that of commercial inorganic detectors, such as silicon [9], gallium arsenide and indium gallium arsenide [10,11]. This discrepancy is primarily due to significant carrier recombination losses, which are further exacerbated when the interface contact is suboptimal. Consequently, the study of interfacial layers in OPDs is of immense scientific significance [12,13].

Generally, there are three primary methods to enhance the interface quality of OPDs: the introduction of hole interface modification layers, such as molybdenum trioxide (MoO_3_) [14], copper thiocyanate (CuSCN) [15], and poly[bis(4-phenyl)(2,4,6-trimethylphenyl)amine (PTAA) [16]; the incorporation of electron interface modification layers, including zinc oxide (ZnO) [17], polyethylenimine (PEI) [18], and bathocuproine (BCP) [19]; and the construction of dipole layers, such as poly(9,9-bis(3-(N,N-dimethyl)-N-ethylammonium-propyl-2,7-fluorene)-alt-2,7-(9,9-dioctylfluorene))dibromide (PFN-Br) and ethoxylated polyethylenimine (PEIE) [18,20,21]. The introduction of hole interfacial layers aims to adjust the anode work function, reduce the hole extraction barrier, block electrons, and prevent interface recombination [22]. Conversely, the incorporation of electron interface layers seeks to lower the cathode work function, reduce the electron extraction barrier, block holes, and enhance electron collection efficiency [23]. The introduction of permanent interface dipoles fundamentally alters the effective work function of the electrode, suppresses trap-assisted recombination, and reduces dark current [24].

Self-assembled interlayers, such as (2-(9H-carbazol-9-yl)ethyl)phosphonic acid (2PACz), represent an excellent class of permanent dipole hole transport materials that have emerged in recent years [25]. These materials demonstrate remarkable capabilities for enhancing performance in fields such as photovoltaics [26,27]. In this study, we further modified the poly(3,4-ethylenedioxythiophene) polystyrene sulfonate (PEDOT:PSS) hole transport layer by incorporating 2PACz, which effectively reduced the dark current of the device while enhancing the cutoff frequency. This improvement is closely related to the dipole effect of 2PACz, which regulates the interface energy levels. Ultimately, the optimized device achieved a specific detection rate of 3.23 × 10^12^ Jones, a rapid response speed of 18 μs, and a broad dynamic response range of 122.8 dB. These metrics surpass those of the control device, which lacks 2PACz, exhibiting a detection rate of 5.56 × 10^11^ Jones, a response speed of 90 μs, and a dynamic response range of 67.9 dB.

## 2. Materials and Methods

**Figure 1:**
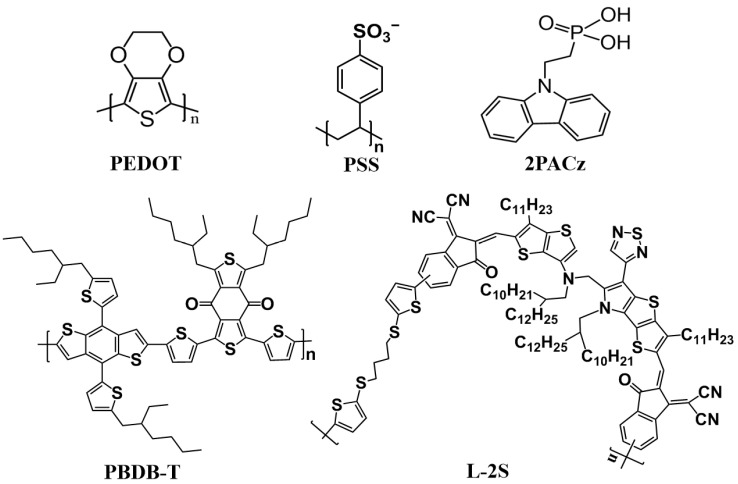
The chemical molecular structures of PEDOT, PSS, 2PACz, PBDB-T and L-2S.

PEDOT:PSS (Xi’an p-OLED), one of the most widely utilized organic conductive materials, was employed as the hole transport layer, while 2PACz (Mreda, Beijing, China), a self-assembled monolayer hole transport material (1–2 nm), served as the hole interfacial layer. Their chemical molecular structures are depicted in Figure 1. The polymer poly[[4,8-bis [5-(2-ethylhexyl)-2-thienyl]benzo [1,2-b:4,5-b′]dithiophene-2,6-diyl]-2,5-thiophenediyl[5,7-bis(2-ethylhexyl)-4,8-dioxo-4H,8H-benzo[1,2-c:4,5-c′]dithiophene-1,3-diyl]] (PBDB-T) and the non-conjugated nonfullerene polymer L-2S acted as the donor and acceptor (DERTHON), respectively, with their chemical molecular structures also illustrated in Figure 1. The PBDB-T:L-2S blend, with a mass ratio of 1:1, was dissolved in chloroform (Guangzhou Chemical Reagents Co., Guangzhou, China) at a total concentration of 13 mg mL^−1^. Meanwhile, 2PACz was dissolved in ethanol (Macklin) at a concentration of 0.27 mg mL^−1^. The electron transport material poly[[2,7-bis(2-ethylhexyl)-1,2,3,6,7,8-hexahydro-1,3,6,8-tetraoxobenzo[lmn][3,8]phenanthroline-4,9-diyl]-2,5-thiophenediyl[9,9-bis[3′((N,N-dimethyl)-N-ethylammonium)]propyl]-9H-fluorene-2,7-diyl]-2,5-thiophenediyl] (PNDIT-F3N) (eFlexPV) was also dissolved in ethanol (with 0.5% acetic acid (Macklin)) at a concentration of 0.27 mg mL^−1^ and subsequently spin-coated onto the active layer at 3000 rpm for 20 s.

The optimized OPD structure is ITO/PEDOT:PSS/2PACz/PBDB-T:L-2S/PNDIT-F3N/Ag, whereas the control OPD structure is ITO/PEDOT:PSS/PBDB-T:L-2S/PNDIT-F3N/Ag, which excludes the self-assembled 2PACz layer. The ITO substrate underwent ultrasonic cleaning in a sequential manner using ethanol, detergent, deionized water, and isopropanol. Subsequently, the substrate was treated with a UV-ozone cleaner for 5 min. The PEDOT:PSS layer was spin-coated onto the pre-cleaned ITO substrate and then annealed at 120 °C for 10 min in ambient air. Following this, the 2PACz solution was spin-coated onto the PEDOT:PSS layer, and the spin-coated active layer was again annealed at 120 °C for 10 min on a hot plate in air. Ag metal was deposited via thermal evaporation under a pressure of less than 4 × 10^−4^ Pa. The effective area of the OPDs is 5 mm^2^. Optical absorption spectra were characterized using a UV-Vis spectrophotometer (UV-2600, SHIMADZU, Tokyo, Japan). Current-voltage (*I*–*V*) curves were recorded with a Keithley source meter (2601A, Keithley, Solonm, OH, USA) under illumination at 780 nm, corresponding to the peak responsivity. The spectral response range was measured with the QE-R external quantum efficiency instrument (Si detector S10-14010, Enlitech, Kaohsiung City, Taiwan). Photoresponse speeds were evaluated by integrating a pulse laser (780 nm) with a digital oscilloscope (Tektronix MSO 3054, Tektronix, Beaverton, OR, USA, 500 MHz). The laser source emits a 780 nm wavelength when connected to a power supply that generates a square wave with a frequency of 100 Hz. The electrical signal output of the device is analyzed under the illumination of the light signal, utilizing a polarizer. Subsequently, the output is connected to an oscilloscope via a wire to visualize the voltage waveform. The on–off ratio curve was recorded by combining a pulse laser (780 nm) with a source meter (2601B, Keithley, Beaverton, OR, USA). Atomic force microscopy (AFM) and Kelvin probe force microscopy (KPFM) images were obtained using the MultiMode AFM (Dimension FastScan, Bruker, Billerica, MA, USA). Contact angle tests for thin films were performed on a DSA100s contact angle goniometer (Lvkeshi, Changzhou Haibei’er Electric Appliance Co., Ltd., Changzhou, China), using water and diiodomethane (DIM) as the testing liquids. Surface energy was calculated with the Owens–Wendt–Rabel–Kaelble method.

Geometry optimizations of all complexes were performed using the B3LYP-D3/6-31G(d) basis set with the Gaussian 16 package [28]. The reduced density gradient (RDG) analysis was conducted to visualize the intermolecular interactions using Multiwfn 3.8 and rendered with VMD 1.9.3 [29]. The binding energies of the complexes were calculated according to the following formula: E_binding energy_ = E(A + B) − E(A) − E(B).

## 3. Results and Discussion

**Figure 2:**
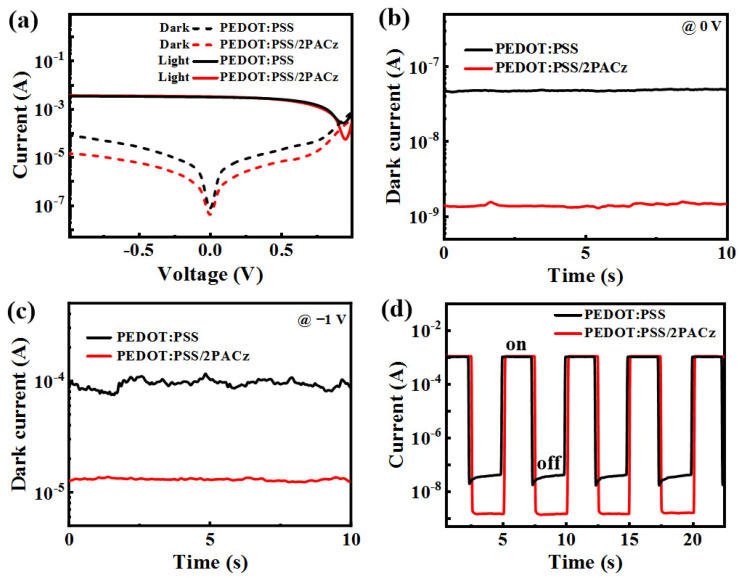
(**a**) *I*–*V* characteristics of two types of OPDs in the dark and under illumination (@780 nm, 1000 mW/cm^2^). The dark current under bias voltages of (**b**) 0 V and (**c**) −1 V for the control and target OPDs, respectively. (**d**) The on–off ratio of the two types of OPDs (@780 nm, 1000 mW/cm^2^).

Figure 2a illustrates the *I*–*V* curves recorded in the dark and under illumination at 780 nm, which corresponds to the peak responsivity (*R*). The photocurrent generated in the target device is comparable to that of the control device; however, the dark current is significantly lower than that of the control device, as evidenced by the diffusion dark current curve at 0 V bias with values of (1.35 ± 0.08) × 10^−9^ A and (4.76 ± 0.08) × 10^−8^ A, respectively (Figure 2b). In OPDs, dark current primarily originates from thermally generated charge carriers and leakage through pinholes/defects at the interface. The reduced dark current in the target device should be attributed to the physical blocking of leakage pathways by the 2PACz interlayer. Furthermore, the open-circuit voltage of the target device exceeds that of the control device under illumination, suggesting the formation of an interface dipole layer following the insertion of 2PACz. Under a negative bias of −1 V, when the external electric field aligns with the built-in electric field, carrier extraction occurs more rapidly. The target device exhibits a more stable and lower drift dark current compared to the control device, with values of (1.4 ± 0.6) × 10^−5^ A and (9 ± 2) × 10^−5^ A, respectively (Figure 2c), indicating that the OPDs utilizing a PEDOT:PSS/2PACz double layer are better suited for high-speed applications, precision measurements, and low light detection.

The current on–off ratio refers to the sensitivity of a photodetector to variations in signal caused by light power under dark and illuminated conditions. A larger value indicates a higher sensitivity of the light response, establishing it as a crucial performance parameter. As illustrated in Figure 2d, the on–off ratios of two types of devices under 0 V and illuminated by a 780 nm near-infrared laser flashing at a cycle of 5 s are presented. The data reveal that the on–off ratio of the target OPD reaches an impressive 7.62 × 10^5^, significantly surpassing the value of 2.63 × 10^4^ for the control device. The stark contrast in off-state behavior can be attributed to the intrinsic ion motion in PEDOT:PSS, which generates a transient current. The PEDOT:PSS/2PACz bilayer serves as an effective interface by physically obstructing this ion migration and optimizing energy levels, leading to a stable, low, and transient-free dark current. This bilayer approach constitutes a robust strategy for enhancing the performance and reliability of OPDs, as it effectively reduces the dark current compared to PEDOT:PSS alone, thereby enhancing the on-off ratio and demonstrating enhanced optical signal recognition capabilities.

**Figure 3:**
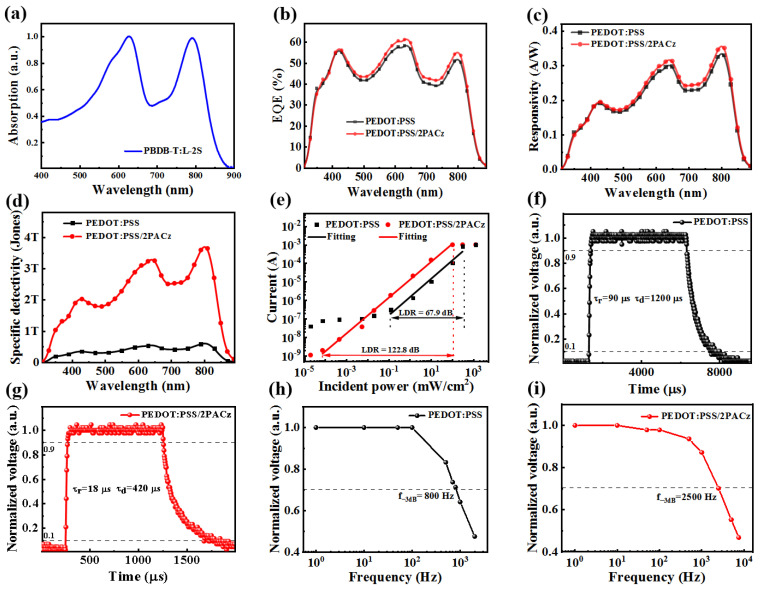
(**a**) The UV-Vis absorption curve of the PBDB-T:L-2S film. (**b**) EQE, (**c**) spectral responsivity at 0 V bias, (**d**) specific detectivity at 0 V bias and (**e**) LDR curves of two types of OPDs at 780 nm. Response speeds of the (**f**) control and (**g**) target OPDs at 780 nm. Frequency responses of the (**h**) control and (**i**) target OPDs at 780 nm.

The absorption spectrum of the PBDB-T:L-2S film is depicted in Figure 3a, showcasing a broad coverage of the visible to near-infrared wavelength region spanning from 400 to 900 nm. Figure 3b illustrates the external quantum efficiency (EQE) of OPDs both with and without the incorporation of 2PACz. An EQE exceeding 40% was achieved across a broad wavelength range from 390 nm to 820 nm, enabling the detection of visible to near-infrared light. Notably, the incorporation of 2PACz resulted in only a marginal increase in the EQE value, which corresponds to *R* of 0.31 A/W compared to 0.29 A/W at 780 nm (Figure 3c). Although the spectral responsivity did not exhibit a substantial increase, the target OPD demonstrates a significantly improved specific detectivity (D*) compared to the control device, with values of 3.23 × 10^12^ Jones versus 5.56 × 10^11^ Jones (Figure 3d), attributed to the markedly reduced dark current. The D* was estimated based on the shot-noise assumption, expressed as D* = *R*/(2q*J*_d_)^1/2^, where *J*_d_ represents the dark current density. We note that if the noise current spectrum were used to calculate D*, the value would be more accurate as it reflects the noise at different frequencies. The linear dynamic range (LDR) of OPDs is illustrated in Figure 3e. The device modified with 2PACz demonstrates a superior linear response of 122.8 dB at 780 nm, in contrast to 67.9 dB for the control device. This finding indicates that the OPD incorporating the interlayer can accurately measure both extremely weak and strong signals simultaneously.

Figure 3f,g illustrate the response times of the OPDs with and without the 2PACz interlayer. The target device exhibits a significantly shorter rise-fall response time of 18 µs and 420 µs under 780 nm laser irradiation, in contrast to the control device, which exhibits response times of 90 µs and 1200 µs, respectively. The observed reduction in response time upon the introduction of the 2PACz interlayer on PEDOT:PSS could be related to several factors. First, 2PACz may form a surface dipole layer on PEDOT:PSS, which might reduce the hole extraction barrier at the interface and facilitate faster charge collection, thereby shortening the carrier transit time [20,21]. Second, the modification with 2PACz could passivate trap states at the interface. By mitigating these traps, charge carriers might be transported and extracted more swiftly, leading to faster rise and decay times [22,23]. Third, the combination of PEDOT:PSS and 2PACz appears to suppress leakage current compared to PEDOT:PSS alone. This suppression may reduce the RC time constant of the device, which is directly related to response speed [24]. Additionally, the target OPD achieves an extensive frequency response of up to 2500 Hz (Figure 3h,i), exceeding the 800 Hz frequency response of the control OPD. This enhanced response speed, coupled with the wide frequency range, facilitates effective monitoring of rapidly varying and high-frequency light signals through the implementation of the interlayer strategy. It is noteworthy that, although quantum efficiency varies with wavelength, the key carrier processes and response speed remain independent of wavelength above the bandgap. Therefore, measurements taken at the 780 nm peak reliably represent the entire broadband response.

**Figure 4:**
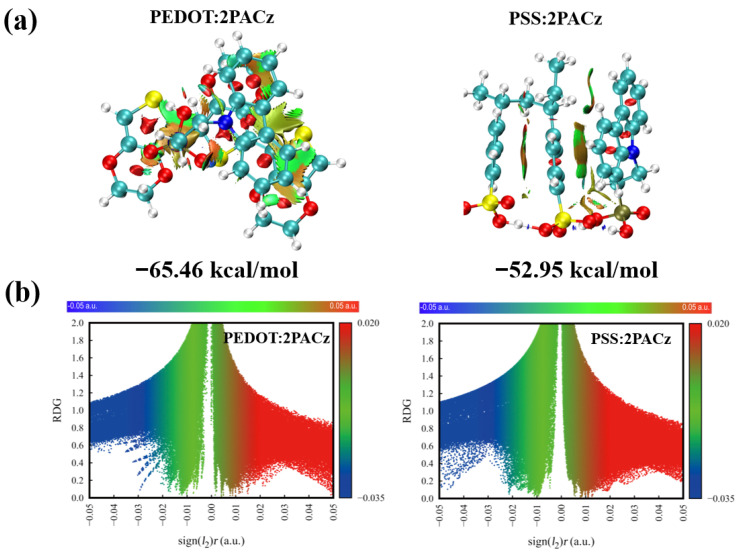
(**a**) RDG isosurface map showing interaction regions for the PEDOT:2PACz and PSS:2PACz complexes with the corresponding non-covalent interaction energy (kcal/mol). (**b**) The diagnostic RDG vs. sign(*l*_2_)*r* scatter plot of the PEDOT:2PACz and PSS:2PACz complexes. In both (**a**) and (**b**), the colors on the RDG isosurface correspond to the colors in the scatter plot, where blue indicates strong attractive interactions (e.g., hydrogen bonding), green indicates weak van der Waals interactions, and red indicates strong non-attractive (steric) interactions.

To clarify the origin of the enhanced OPD performance at the molecular level, we conduct a theoretical analysis of the intermolecular interactions between PEDOT and 2PACz, as well as PSS and 2PACz in the gas phase (Figure 4). As illustrated in Figure 4a, the complex system of PEDOT and 2PACz exhibits attractive interactions that primarily consist of π-π interactions between the conjugated systems of the two planar molecules, alongside hydrogen bonding interactions. These hydrogen bonds form between the C=O or O-H groups at the phosphate end of 2PACz and the C-H, O, or S atoms within PEDOT, with no significant repulsive interactions detected. In the case of the complex system involving PSS and 2PACz, the attractive weak interactions predominantly include π-π stacking between the two benzene rings of PSS, π-π stacking between PSS and 2PACz, and hydrogen bonding between the sulfate group of PSS and the phosphate group of 2PACz. The interaction energy between PEDOT and 2PACz is significantly negative (−65.46 kcal/mol), indicating enhanced thermodynamic stability of the system, which facilitates the formation of a more stable interfacial layer. Conversely, the negative interaction energy between PSS and 2PACz is comparatively smaller (−52.95 kcal/mol), suggesting that the system is more prone to dissociation and exhibits lower charge transfer efficiency.

Figure 4b illustrates the diagnostic RDG scatter plot of the PEDOT:2PACz and PSS:2PACz complexes. The PEDOT:2PACz system exhibits a highly concentrated and dark blue iso-surface, indicating strong electrostatic interactions between PEDOT and 2PACz. In contrast, the PSS:2PACz system displays lighter and more dispersed blue regions, suggesting that PSS:2PACz is primarily influenced by multiple hydrogen bonding and dispersion interactions, albeit with limited strength for each interaction. This theoretical analysis elucidates a fundamental mechanism underlying the superior performance of PEDOT:2PACz in OPDs. The 2PACz molecule serves as a bridge, infiltrating the PEDOT:PSS film with the aid of a solvent. It effectively locates PEDOT molecules to form bonds. This process reconstructs a new interface that is more conducive to hole transport, enriched in PEDOT, and tightly coupled with the upper 2PACz layer [25,30]. Essentially, when 2PACz is layered on the surface of PEDOT:PSS, a stronger and more directional electrical coupling is established between 2PACz and PEDOT:PSS.

**Figure 5:**
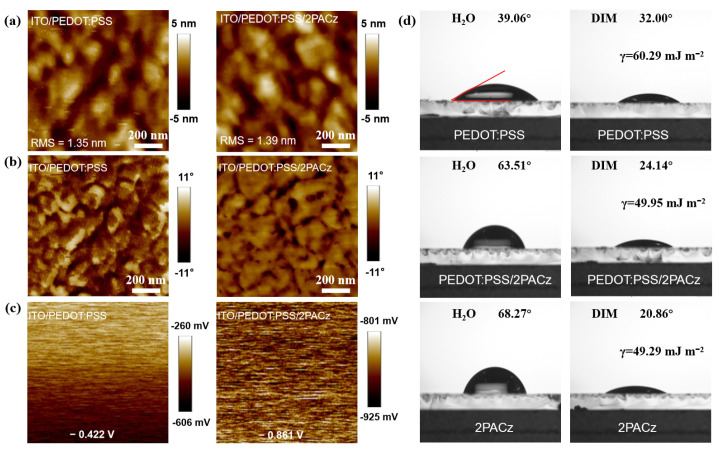
(**a**) AFM height, (**b**) AFM phase and (**c**) KPFM images of the PEDOT:PSS and PEDOT:PSS/2PACz films (scan area = 1 μm × 1 μm). (**d**) Contact angles of the PEDOT:PSS, PEDOT:PSS/2PACz and 2PACz films.

To experimentally verify the interaction between PEDOT:PSS and 2PACz, we examined changes in morphology and work function. Figure 5a illustrates the surface morphologies of the films (PEDOT:PSS and PEDOT:PSS/2PACz) assessed via AFM. The morphologies of PEDOT:PSS and 2PACz deposited on PEDOT:PSS are relatively smooth, with root-mean-square (RMS) roughness values of 1.35 nm and 1.39 nm, respectively. However, the 2PACz on PEDOT:PSS exhibited fewer deep valley features, as observed in the phase images (Figure 5b), indicating that 2PACz can effectively fill these surface defects and reduce leakage current. Moreover, the surface potential of PEDOT:PSS is −0.422 V, while that of PEDOT:PSS/2PACz is −0.861 V (Figure 5c). This suggests that the monolayer 2PACz layer functions as an electric dipole layer (with the phosphate terminal being negative and the PEDOT:PSS being positive), resulting in a downward shift in the work function from −5.0 eV to −5.4 eV. This shift significantly decreases leakage current, enhances photocurrent generation, and improves the light response speed of the device. Additionally, these changes are reflected in the alterations in the surface energy of the films (Figure 5d). The PEDOT:PSS surface is rich in PSS, which contains strongly polar -SO_3_^−^ groups and unpaired oxygen atoms, providing strong dispersion and polarity forces that lead to an increase in surface energy. 2PACz is anchored to the surface of PEDOT through phosphate groups. Consequently, the surface is exposed to non-polar carbazole groups and connected alkyl chains/aromatic ring organic frameworks, resulting in significantly reduced polarity. Furthermore, this reduction in surface energy is highly beneficial for the formation of subsequent organic layers.

## 4. Conclusions

In conclusion, this study presents a straightforward approach to enhancing the performance of visible/near-infrared OPDs by incorporating an interlayer of 2PACz to modify the properties of PEDOT:PSS. The optimized OPD demonstrates a lower dark current, a higher switching ratio, increased responsivity, and an improved specific detection rate compared to the control device based on PEDOT:PSS. Furthermore, the response time, cutoff frequency, and linear dynamic range have been significantly enhanced. This improved performance may be attributed to the formation of a new PEDOT-rich interface that facilitates hole transport and is tightly integrated with the upper 2PACz layer. This study highlights considerable potential for OPD performance enhancement through hole interface engineering.

## Data Availability

The data that support the findings of this study are available from the corresponding author upon reasonable request.
